# Optimal placement of flexible ureteral access sheath in retrograde intrarenal surgery

**DOI:** 10.1007/s00240-023-01469-9

**Published:** 2023-08-17

**Authors:** Yujun Chen, Xiaofeng Cheng, Heng Yang, Wen Deng, Luyao Chen, Gongxian Wang, Xiaochen Zhou

**Affiliations:** Department of Urology, The First Affiliated Hospital of Nanchang University, 17 YongWai Street Surgery Building, 17th Floor, Nanchang, 330006 Jiangxi China

**Keywords:** Ureteral access sheath, Retrograde intrarenal surgery, Ureteroscopy, Stone fragments

## Abstract

This study aims to explore the optimal location of flexible ureteral access sheath (f-UAS) in retrograde intrarenal lithotripsy (RIRS). RIRS model was built by AutoCAD 2011 software, and imported COMSOL 5.6 software to computer simulation. An RIRS model was constructed in vitro to analyze the distribution pattern of stone fragments and compare the weight of stone fragments carried out by the irrigation fluid when the f-UAS is in different positions. Computer simulation showed that the highest flow of irrigation fluid was in the channel of flexible ureteroscopy (f-URS) and in the lumen of f-UAS. From the f-URS to the renal collection system and then to the f-UAS, the velocity of irrigation fluid changes gradually from high-flow to low-flow and then to high-flow. When the f-URS and the f-UAS are at the same level, the irrigation fluid is always at a state of high flow during the process from f-URS to f-UAS. When the f-URS and the f-UAS are at the same level, it can increase the local intrarenal pressure (IRP) at the front of f-URS. The stone fragments are mainly sediment in the low-flow region of irrigation fluid. More stone fragments could follow the irrigation fluid out of the body when the tip of f-URS and the tip of f-UAS are at the same level (*P* < 0.001). The f-UAS should be brought closer to the stone in RIRS. And more stone fragments can be taken out of the body by the effect of irrigation fluid.

## Introduction

The ureteral access sheath (UAS) facilitates flexible ureteroscopy (f-URS) exercise, reduces operative time, lowers intrarenal pressure (IRP), and improves stone-free rate (SFR) in retrograde intrarenal surgery (RIRS) [1–4]. There are no uniform guidelines for the placement of the traditional UAS, most traditional UAS are placed in the upper ureter and not in the renal pelvis, much less so in the calyces [5]. A joint consensus recommends placing the traditional UAS 2 cm below the ureteropelvic junction (UPJ) [5]. The UAS may be blocked by the ureteral mucosa and interfere with the outflow of irrigation fluid when placed in the upper ureter, ultimately leading to an increase in IRP [6, 7]. Many powdered or granular stones are produced during RIRS, and these stone fragments cannot be removed by lithotripsy baskets and can only be removed by self-elimination postoperatively [8, 9]. Renal colic and hematuria often occur during the self-elimination process [10, 11]. With the development of technology, a novel flexible ureteral access sheath (f-UAS) has been successfully developed and used in the clinic with good surgical results [12]. The f-UAS is a new type of UAS that has a tip of 10 cm tube that can be passively bent with the bending of f-URS, it can be placed in the renal pelvis or calyces, and can actively control IRP and obtain a complete SFR [12, 13]. With advances in technology, some consensus may be changed, so when f-UAS is in that position can better control IRP and improve SFR?

A computerized model of RIRS was established to analyze the distribution pattern of irrigation fluid in RIRS. And vitro RIRS model was established to analyze the distribution pattern of stone fragments under the action of irrigation fluid. Compare the weight of stone fragments carried out by the irrigation fluid. We inferred the optimal placement of f-UAS in RIRS.

## Materials and methods

### A computerized model of RIRS

The RIRS model was created using AutoCAD 2011 software and then imported into COMSOL 5.6 software for computer simulation. The irrigation fluid was set at 80 ml/min. A two-dimensional model was generated to analyze the distribution pattern of irrigation fluid when the f-URS position was stationary in renal calyces, and the f-UAS was in the upper ureter or renal pelvis.

### Vitro model of RIRS

An RIRS model was established using a tube (10 ml), f-UAS (12/14F, 35 cm; ZHANGJIAGANG, Jiang Su, China), and f-URS (8.6 Fr; ZebraScopeTM, China). Stone fragments (200 mg, less than 2 mm) were placed at the bottom of the tube. The irrigation fluid was set at 80 ml/min. We analyzed the distribution pattern of stone fragments under the action of the irrigation fluid when the f-UAS was in different positions.

Flexible ureteral access sheath (f-UAS): the f-UAS is a new type of UAS that has a tip of 10 cm tube that can be passively bent with bending of flexible ureteroscope (f-URS), and this degree of bend is about 145º. The f-UAS is still able to ensure a positive circular shape of tube lumen when bending, and it can be connected to a vacuum suction device [13].

A group: the f-UAS and the f-URS are basically at the same level, and both move synchronously and gradually approach the stone fragments. B group: the f-URS was ≥ 2 cm away from the f-UAS, the f-URS was in constant motion, and the f-UAS was fixed (B Group simulates that most of the traditional UAS can only be placed in the UPJ, when the UAS are at some distance from the f-URS that lithotripsy in the renal.). The experiment was conducted for 10 min in both groups and the weight of stone fragments carried out by irrigation fluid was compared between the two groups. Repeat 20 times each in two groups.

Student’s *t* test was applied to continuous data. Statistical significance was set at *P* < 0.05.

## Result

Computer simulation showed that the highest flow of irrigation fluid is in the channel of f-URS and in the lumen of the f-UAS, which is a region with the relatively highest flow. A region with a relatively low flow of irrigation fluid has been reported elsewhere. During the process in which the irrigation fluid from the f-URS to the renal collection system and then to the f-UAS, the velocity of the irrigation fluid changes gradually from high-flow to low-flow and then to high-flow.

**Fig. 1:**
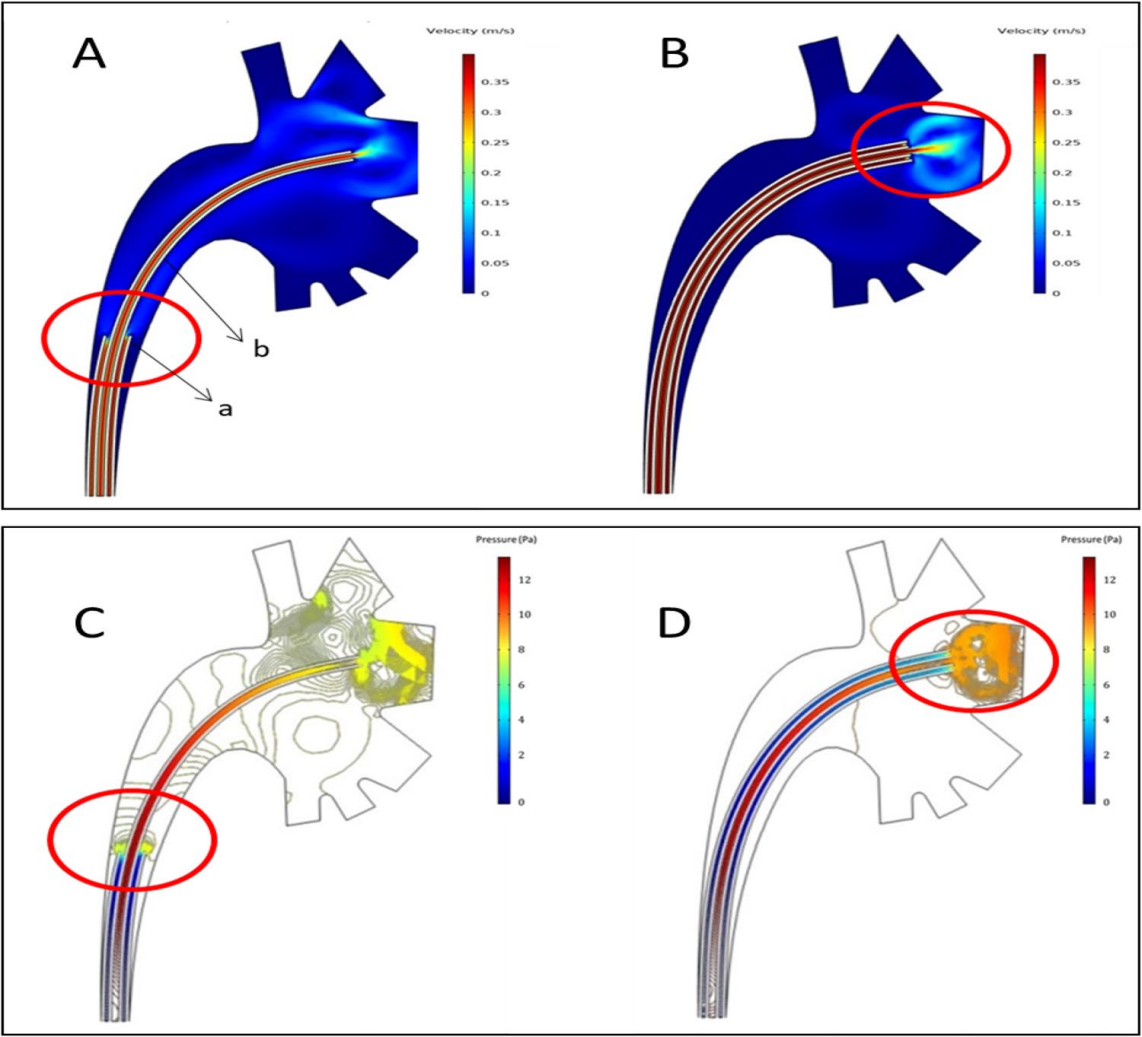
Computerized model of RIRS: distribution pattern of the velocity of irrigation fluid (**A**, **B**) and the intrarenal pressure (**C**, **D**). Flexible ureteral access sheath (f-UAS) in upper ureter (**A**, **C**); f-UAS in renal pelvis (**B**, **D**). a: f-UAS; b: flexible ureteroscopy

When the tip of the f-URS and the tip of the f-UAS are at the same level, the irrigation fluid is always in a state of high flow during the process from f-URS to f-UAS, and there is almost no low-flow state. Comparing the two situations where the f-UAS is in different positions, it was found that a localized higher IRP is generated at the tip of the f-URS when the tip of the f-URS and the tip of the f-UAS are at the same level (Fig. 1).

**Fig. 2:**
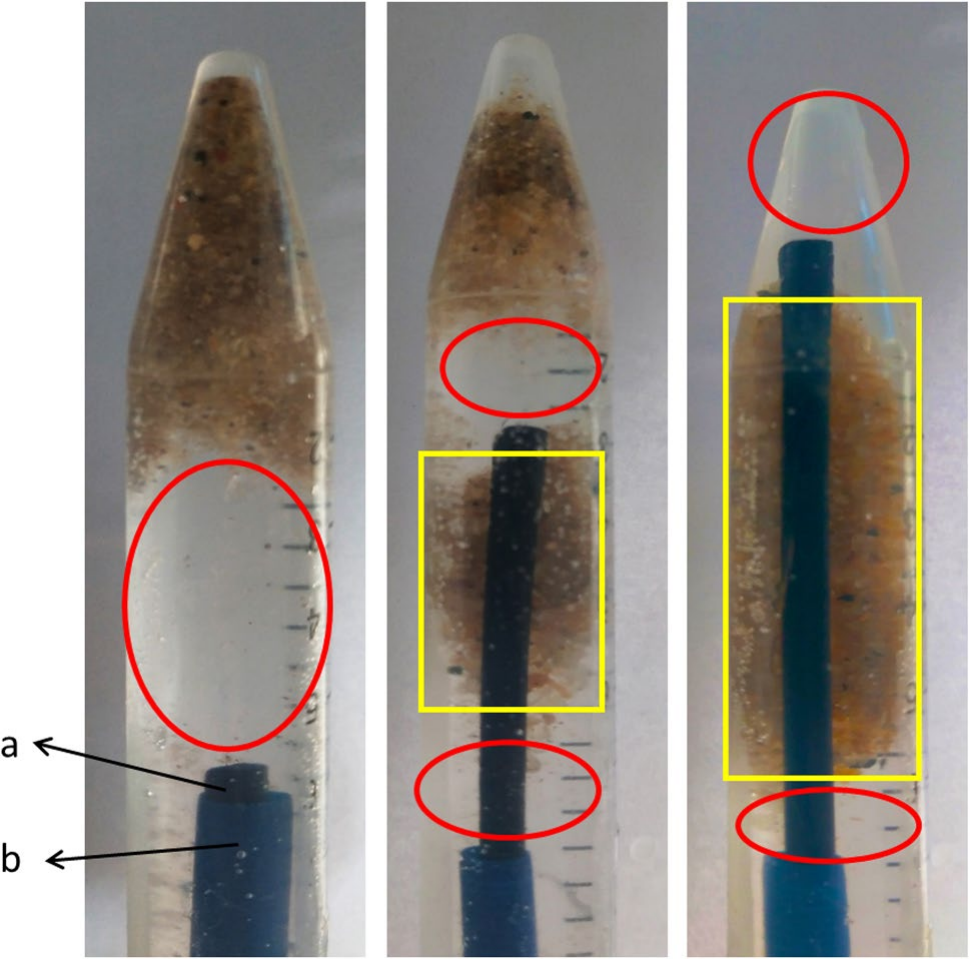
Vitro model of RIRS: The distribution pattern of stone fragments under the action of the irrigation fluid when the flexible ureteral access sheath (f-UAS) was in different positions. a: flexible ureteroscopy; b: f-UAS

Observed in conjunction with Fig. 1, the vitro model results show that stone fragments sediment mainly in the low-flow region of irrigation fluid (Fig. 2: yellow frame), and stone fragments do not sediment in the high-flow region of irrigation fluid (Fig. 2: red circle). More stone fragments could follow the irrigation fluid out of the body when the tip of the f-URS and the tip of the f-UAS are at the same level and close to the stone fragments (*P* < 0.001) (Table 1).

**Table 1:** The weight of stone fragments carried out by irrigation fluid

	A Group (200 mg)	B Group (200 mg)	*P* value
1	67	23	< 0.001
2	63	30	
3	69	25	
4	58	24	
5	59	18	
6	66	23	
7	72	24	
8	76	19	
9	68	25	
10	72	27	
11	71	21	
12	68	28	
13	64	23	
14	68	28	
15	71	32	
16	69	37	
17	63	29	
18	67	26	
19	71	32	
20	62	24	

## Discussion

With advances in technology, a variety of UAS has been invented and used in clinical applications [12, 14, 15]. Some consensus may be outdated or changed. In this study, we have used computer simulations and in vitro experiments to reveal the distribution pattern of irrigation fluid and IRP when the f-UAS is in different positions, and to compare the weight of stone fragments that can be carried out under the action of the irrigation fluid. Our results showed that the fastest velocity of irrigation fluid is in the lumen of f-URS and f-UAS, which is a region with the relatively highest flow. A region with a relatively low flow of irrigation fluid is elsewhere. The velocity of irrigation fluid is close to zero in some regions. During the process in which the irrigation fluid from the f-URS to the renal collection system and then to the f-UAS, the velocity of irrigation fluid changes gradually from high-flow to low-flow and then to high-flow. As the two high-flow regions gradually approach, or it can also be referred to as the end of f-URS and the end of f-UAS gradually approach, the low-flow region gradually decreases or disappears.

Stone fragments are constantly moving under the action of irrigation fluid, but at the same time, the stone fragments are also subjected to the action of gravity, which eventually leads to sedimentation. Observed in conjunction with Fig. 1, the vitro model results show that stone fragments sediment mainly in the region of low-flow of irrigation fluid, and stone fragments do not sediment in the region of high-flow. The low-flow region can be referred to as the region of sediment for stone fragments. The low-flow region gradually decreases or disappears when the f-UAS is at the same level as the f-URS, so more stone fragments are taken out of the body by the action of irrigation fluid when the tip of the f-URS and the tip of the f-UAS are at the same level and close to the stone fragments (*P* < 0.001).

When the f-UAS and f-URS are at the same level, only a localized high pressure is generated at the front of the f-URS, rather than an increase in intrarenal pressure throughout the renal collecting system. In our opinion, one of the reasons for high intrarenal pressure is the presence of some kinetic energy in the irrigation fluid. In addition, in the computer model, the f-URS is equivalent to being placed in the calyx, and a certain thickness of the sheath exists, which indirectly reduces the outlet of the calyx when the f-UAS is also placed in the calyx, i.e., reduces the outlet of the irrigation fluid. This eventually leads to an increase in IRP. The IRP can be reduced by connecting a vacuum device; an f-URS and a UAS at the same level and connected to a vacuum device might better improve the stone free rate and reduce intrarenal pressure [12–16].

To understand the effect of irrigation fluid on stones, we introduced the concept of the drag force (*F*_D_) in fluid dynamics. *F*_D_ is one of the forces on the sediment particles by the current [17]. *F*_D_ equation: ${F}_{\mathrm{D}}={C}_{\mathrm{D}}A{D}^{2}\frac{\uprho {{U}_{\mathrm{b}}}^{2}}{2}$ (${U}_{\mathrm{b}}$: instantaneous velocity of current; *C*_D_: drag and lift coefficients; *A*: frontal area exposed to the flow; ρ: densities of fluid and sediment) [17]. *F*_D_ is proportional to ${{U}_{\mathrm{b}}}^{2}$. This indicates that the *F*_D_ increases exponentially with the increase in current velocity, which causes the stone fragments to move more easily with the irrigation fluid. The irrigation fluid is always at a high-flow rate from f-URS to f-UAS when f-UAS is at the same level as f-URS, so more stone fragments are taken out of the body by the action of irrigation fluid. The velocity of irrigation fluid cannot be increased indefinitely in RIRS, but bringing the high-flow region closer to the position of stone fragments can indirectly increase the action of irrigation fluid on stone fragments. However, the irrigation fluid flow rate is influenced by several factors [18]. A vacuum cleaner effect is often described in percutaneous nephrolithotripsy (PCNL). The puncture sheath is almost vertical in PCNL; however, stone fragments can overcome gravity and be carried out of the body by the action of the irrigation fluid. Mager, Zeng and Nicklas have also shown that the irrigation can create turbulence or a vortex at the distal end of the endoscopic in PCNL; turbulence and vortices create a vacuum cleaner effect, which results in more stone fragments being carried out of the body [19–21]. Our computer model shows that the irrigation fluid at the front of f-URS produces turbulence and vortices. Therefore, we think there is also a vacuum cleaner effect on the front of the FURS. In summary, we think that when the f-UAS and f-URS are at the same level and gradually approaching the stone fragments, more stone fragments can be taken out of the body by the action of the irrigation fluid and vacuum cleaner effect.

Powdered and granular stones are inevitably produced during RIRS [11, 22, 23]. Some studies have shown that the residual rates of stone fragments < 3 mm, < 2 mm, and < 1 mm after RIRS were 10–15%, 16. 1% and 86%, respectively [24, 25]. Our study has shown that when the f-UAS is brought closer to these stone fragments, it can bring significantly more stone fragments (< 2 mm) out of the body. This may be a way to improve the SFR. Chen et al. used a novel flexible UAS closer to the stone in RIRS and ultimately obtained a better SFR, our results are consistent with theirs [12, 13].

The experiments showed that more stone fragments could be carried out of the body under the action of irrigation fluid when the UAS is at the same level as the f-URS and both move synchronously. In reality, it is difficult for the non-flexible UAS to follow the f-URS into the renal pelvis or calyces. For non-flexible UAS, perhaps the optimal location in ureteral stones and bladder stones may also be close to the stone. However, this study was only an in vitro experiment and computer simulation without the influence of devices such as a laser; this does not represent the true in vivo situation, which requires further clinical validation.

## Conclusion

During the process in which the irrigation fluid from the f-URS to the renal collection system and then to the f-UAS, the velocity of irrigation fluid changes gradually from high-flow to low-flow and then to high-flow. Stone fragments were mainly sediment in the low-flow region of irrigation fluid. More stone fragments could follow the irrigation fluid out of the body when the f-UAS is at the same level as the f-URS and both move synchronously and progressively closer to the stone fragments.

## Data Availability

The authors confirm that the data supporting the findings of this study are available within the article.
